# Brief migraine episodes in children and adolescents-a modification to International Headache Society pediatric migraine (without aura) diagnostic criteria

**DOI:** 10.1186/2193-1801-2-77

**Published:** 2013-03-04

**Authors:** Muttamthottil Varghese Francis

**Affiliations:** Headache & Neuroophthalmology Services Teresa Eye and Migraine Centre, Cherthala, Alleppey, Kerala 688527 India

**Keywords:** Brief migraines, ICHD2, Modification, Common migraine triggers family history

## Abstract

The international Headache Society (I H S) diagnostic criteria (International classification of headache disorders edition 2- ICHD 2) for headache in children and adults improved the accuracy of migraine diagnoses. However many short duration headaches in children, receive an atypical migraine diagnosis. This study is to diagnose children and adolescents who presented with such atypical migraines of less than one hour duration. 1402 children and adolescents aged 5 to 15 years who presented with recurrent brief activity affected head pain, were studied. Known and common migraine triggers and family history of migraine were recorded in all. All the children studied had moderate to severe headache lasting 5 to 45 minutes which forced them motionless during the attacks (thus fulfilling 2 diagnostic pain features). At least one of the ICHD2 pediatric migraine diagnostic symptoms (nausea / vomiting / photophobia / phonophobia) were present in all. Two additional features were diagnostic of brief migraines in all of them- one of the parents or siblings was a migrainer and one of the common migraine triggers as a precipitating factor. This study concludes that if duration of head pain is less than one hour ,two additional features to be included to diagnose definitive migraine in children and adolescents - one migraine parent or sibling and one of the migraine triggers precipitating the head pain.

## Introduction

Prior to the publication of I H S Headache classification system in 1988 ( Headache classification committee of The International Headache Society 1988 ), several classifications were used for headache diagnosis in children. These dealt mainly with migraine and included the criteria established by Vahlquist ( 1955 ), Deubner ( 1977 ) and Prensky and Sommer ( 1979 ). During the last 15 years, several studies ( Seshia and Wolstein 1994 ; Metsahonkala and Sillanpaa 1994 ; Mortimer et al. 1992a ; Wober Bingol et al. 1996 ; Francis 2002 ) have proposed revisions to the I H S criteria for children and adolescents with migraine. The major suggestions were to shorten the duration of the migraine attack to one hour and to remove hemicrania as a criterion since many children have headaches that are bi temporal or bi frontal. An additional suggestion was to require either photophobia or phonophobia instead of both ( Winner et al. 1995 ).

However, when I H S criteria and the various modifications are applied, a sizable proportion of headache children with migrainous features fail to fully meet I H S migraine criteria and thus they receive an atypical migraine diagnosis ( probable migraine according to ICHD 2 2004 ). Many children report brief episodes of head pain which resemble migraine but these migraine episodes of less than one hour duration are not well documented. Little is known about the frequency of these head pain attacks in the childhood and adolescent age groups. Moreover there are overlapping statements in the diagnostic criteria of migraine and tension type headaches- number of episodes, duration of pain, bilaterality, moderate intensity and either phonophobia or photophobia- all these features are common for making both diagnoses.

Present study is undertaken to document brief migraine episodes lasting less than one hour duration in children and to suggest additional features to distinguish migraine from tension type headaches and to propose a modification to pediatric migraine (without aura) diagnostic criteria.

## Methods

9620 children and adolescents aged 5 to 15 years who presented with recurrent short duration head pain of less than 2 hours at The Teresa Eye and Migraine centre and St Sebastians Visitation Hospital, Arthunkal in Cherthala, Alleppey were studied prospectively, spanning 4 years. Children attending the monthly charity headache camps also included. The inclusion criteria were designed from the authors 15 years experience ( Francis 2002 , 2004 ) in managing nearly 30,000 headache children prior to this study. The inclusion criteria were recurrent head pain (minimum 5 episodes) of less than one hour duration, activity affected (motionless) during the head pain episodes, one ICHD 2 diagnostic associated feature (nausea / vomiting / phonophobia / and or photophobia), one common migraine trigger precipitating the attacks and one parent or sibling suffering from ICHD 2 Migraine (with or without aura) headaches. Information regarding the duration, severity, quality and location of head pain and behaviour during head pain episodes were also recorded. Children with visual manifestations, typical ICHD2 tension type headaches and other headaches of eye, ENT and dental origin were excluded so also fever and other systemic and organic illnesses.

## Results

1402 children and adolescents fulfilled the inclusion criteria to diagnose brief migraine episodes of less than one hour duration (5 minutes to 45 minutes). There were 842 girls and 560 boys. The headache characteristics, common migraine triggers and family history of 1402 children are given below.

### Headache characteristics

Duration - 5 to 15 minutes 112(8%), 15 to 30 minutes 211(15%), 30 to 45 minutes 1079(77%).

Location - always unilateral 448(32%), bilateral 785(56%), unilateral spreading to bilateral 169(12%).

Quality - pulsating 518(37%), non pulsating 617(44%), just ache(not able to explain) 267(19%).

Behaviour during attack- sit quiet 588 (42%), lie down (with or without pressing on temples) 449(32%), applying balm and sleep off 365(26%).

Associated features- nausea 252(18%), vomiting 196 (14%), phonophobia 883(63%) and photophobia 798(57%).

### Common migraine triggers

exposure to sunlight 1290(92%), travelling by bus 673(46%), strenous physical exercises like dancing and cycling 590(42%), sleep disturbances 336(23%), missing meal at the right time 296(21%).1010(72%) reported more than one trigger.

Tension anxiety situations like examinations and funerals were another significant common trigger but omitted in this study, not to confuse with tension type headaches.

### Family history

mother-1148 (82%)

father-155 (11%)

siblings-99 (7%)

DIAGNOSTIC CRITERIA of migraine without aura

ICHD 2

5 attacks

 Duration 1 to 48 hours

Pain characteristics (2 of 4)

 Either bilateral or unilateral

 Pulsating quality

 Moderate to severe intensity

 Aggravated by routine activities

Autonomic symptoms (associated features) (1 of 3)

 Nausea / vomiting / Photophobia and phonophobia

(ICHD 2 recommends both photophobia and phono-phobia for diagnosis and for untreated duration of less than 2 hours, corroboration by prospective diary studies).

Episodic tension type headache (ICHD 2)

### Diagnostic criteria

10 episodes

 duration -30 minutes to 7 days

 2 of 4 pain characteristics

  Bilateral location

  Non pulsating quality

  Mild or moderate intensity

  Not aggravated by routine physical activities

 Autonomic symptoms or associated features

 Both of the following

  No nausea or vomiting

  No more than one of phonophobia or photophobia

## Discussion

Studies show that in children and adolescents, migraine tends to be of shorter duration. The duration of head pain was reported to be less than 2 h in 11–81% and less than one hour in 8 to 25% in different studies ( Maytal et al. 1997 ; Mortimer et al. 1992b ). Similarly Metsahonkala ( 1994 ) reported that when duration was omitted as a criterion the prevalence of migraine increased by 25.9%. In fact Gherpelli and colleagues ( 1998 ) found that entirely excluding duration criterion increased the sensitivity without decreasing the specificity of pediatric migraine diagnosis. This study supports the suggestion of decreasing the criterion on the minimal duration of head pain to less than one hour for migraine without aura in children.

In this study, 1402 children reported recurrent activity affected head pain lasting 5 to 45 minutes with one of the associated diagnostic migraine features of nausea / vomiting / phonophobia / and or photophobia. It was difficult to get the symptoms of both phonophobia and photophobia from majority of these children at first consultation and interviewing both the parents, family members and class mates were necessary in eliciting both the symptoms. This is usually not possible and practical in countries like India, where huge volumes of patients (up to 250 /day) have to be examined on a daily basis. The behaviour during head pain episodes like switching off TV and radio, closing the door and putting off lights, covering the heads with blanket or cloth when lying down with head pain, getting angry or shouting at family members or classmates when they try to talk etc. were indirect evidences suggestive of both.

All of them were getting the headpain attacks when exposed to one or more of the common migraine triggers ( Francis 2002 , 2004 , 2009 ) in this region. Exposure to sunlight and traveling by bus were the most common triggers. Mortimer et al. ( 1992b ) reported that a migraine trigger could be identified in 44.4% of the children aged 8–11 years. In children more than 8 years tiredness, exercise, noise, glaring light, missing a meal were all reported as migraine precipitants by different studies. Rossi et al. ( 1989 ) documented psychological stress followed by physical stress as the commonest precipitating factors in childhood migraines. This is the first study to document common migraine triggers in a region to aid in the migraine without aura diagnostic work up. Majority of the children and their parents reported same common triggers with exposure to sunlight precipitating migraine in nearly 90% of them. Trigger factors are less common or less obvious in patients with episodic tension type headaches. It is documented that emotional stress, lack of sleep ( Blau 1990 ) and menstruation can trigger or aggravate both TTH and migraine but activity getting affected, other common migraine triggers precipitating pain and family history will favour a diagnosis of migraine in such clinically confusing scenario ( Francis 2009 ). This study didn’t consider stress and menstruation as triggers and lack of sleep is found to be one of the very common triggers for ICHD2 migraine in this region of India.

Family history revealed mother (82%), father or sibling suffering from ICHD2 migraine with or without aura. Migraine is a familial disorder, although disagreement exists regarding the mode of inheritance. If one looks at the families of children with migraine, 50 to 90% of relatives also have migraine. Parents must be questioned in detail to find out migraine symptoms. Most of them considered their headaches are different from what their children are getting. The diagnoses as told to them by their medical practitioners (the first contact practitioner in their life) are sinus, low (especially if dizzy spells are associated with headaches) or high blood pressure, tension, spectacle related, ear balance dysfunction especially when dizziness or vertigo associated with recurrent headaches, anemia, vitamin deficiency or functional. Therefore leading questions like whether they get headache when exposed to sunlight, bus traveling or other known or common migraine triggers must be specifically asked to unravel migraine symptomatology. It was indeed surprising to find out that some parents considered head throbbing, severe head discomfort and head pain as different entities. Many mothers thought that sun exposure headaches are normal ordinary headaches and there is no need to mention about it to the doctor. When details of these headaches were asked typical migraine features were revealed.

This study shows that reducing the time duration to less than one hour would considerably increase the number of children diagnosed with migraine. One can argue that this time reduction might increase the overlap between the diagnostic criteria of migraine and tension type headaches but it can be easily overcome by adding one common/known migraine trigger and one family member suffering from ICHD2 migraine to the present diagnostic features. One cannot consider any other diagnoses in these children even if only one of phonophobia or photophobia is present. Other short duration activity affected headaches like cluster headaches and paroxysmal hemicranias, though reported in children, are very rare and no case was diagnosed in this age group during the study period. Short duration mild to moderate non throbbing headaches attributed to uncorrected refractive errors, phorias and tropias were diagnosed when prolonged and tiring near or far focusing precipitated peri orbital pain. Other brief headaches attributed to cough, valsalva, cold, external compression etc. were easy to diagnose clinically and was not a problem differentiating from brief migraine without aura episodes. Children and adolescents complaining of aura like manifestations were not included in this study and benign occipital epilepsies with brief colored auras with automatisms were not a diagnostic consideration in these patients.

A critical analysis of the I CHD2 diagnostic criteria for migraine and tension, exposes more than one overlapping statements. In this study, majority of the children presented with bilateral (68%) non throbbing (63%) headaches (this fulfills two diagnostic pain features for tension type headaches) and with the duration of more than 30 minutes and one associated feature (phonophobia or photophobia) one tends to diagnose episodic tension type headaches in these children (missing two criteria to diagnose ICHD 2 migraine without aura). At the same time probable migraine (missing duration criterion) ( Granella et al. 1994 ; Rasmussen et al. 1991 ) too can be diagnosed because of activity affected moderate to severe intensity head pain and one associated feature. In these clinically confusing situations the following three features clearly differentiate migraine from tension type headaches. 1) activity affected head pain (motionless) 2)one common / known migraine trigger precipitating pain 3)one family member suffering from I CHD 2 migraine (definite or probable). The problem in diagnosing probable migraine is that, most of the parents are concerned about an underlying brain tumor or other serious disease and one can confidently explain to them that what their children are getting is nothing but brief migraine attacks. Pointing out the triggers and family history with a negative general, physical and neurological and neuroocular exam will be very reassuring and scientifically more convincing to both children and family.

Thus this study shows that both migraine and tension can be distinguished easily from a thorough clinical history. Therefore it is proposed that brief migraine attacks to be diagnosed in children and adolescents with less than one hour duration and must be differentiated from episodic tension type headaches. ICHD2 to be modified as - if duration of head pain is less than one hour, two additional features to be added to diagnose migraine without aura in children.

1. one common/known migraine trigger precipitating the attacks.
2. one parent or sibling suffering from I CHD2 migraine (definite or probable).

## Ethics

This study proposal was approved by the hospital ethics committee.

## Data sharing

There is no other unpublished data to share.

## Design

Prospective cohort study. Setting: Teresa Eye and migraine centre, Cherthala and St Sebastians Visitation Hospital, Arthunkal, Kerala.

## Funding

This research project was not funded by any group or institution.

## Objective

To diagnose brief migraine episodes in children and adolescents and to propose a modification to pediatric migraine without aura diagnostic criteria of the International headache society.
